# Small family cheesemaking in Mexico: Microbial quality and good manufacturing practices

**DOI:** 10.3168/jdsc.2025-0801

**Published:** 2025-10-10

**Authors:** Luis A. Ibarra-Sánchez, Alan L. Olvera-Aguilar, Kruskaia K. Caltzontzin-Fernández, José A. Cabrera-Luna

**Affiliations:** Facultad de Ciencias Naturales, Universidad Autónoma de Querétaro, Querétaro, 76230, México

## Abstract

Small family dairy farmers are a type of production system in Mexico characterized by family-owned farms where family members are dedicated to the production of milk and dairy products such as cheese. Artisanal cheeses made by small family cheesemakers are typically made with raw milk, raising concern for presence of spoilage and pathogenic microorganisms. Although small family cheesemakers have traditional intergenerational knowledge of cheese manufacturing, their knowledge of hygienic practices may be limited. This mini-review provides an overview of the microbial quality of artisanal cheeses made by small family cheesemakers in Mexico and their manufacturing practices as factors affecting cheese microbial contamination.

## Highlights

• Most artisan cheeses made by SFC in Mexico had poor microbial quality.
• Raw milk Cocido cheese had acceptable microbial quality under Mexican regulations.
• Cheese manufacturing and hygienic practices also contribute to cheese contamination.
• Some SFC heat treat raw milk, but this did not improve cheese microbial quality.
• Training in good manufacturing practices may help SFC to improve cheese safety.

## Graphical Abstract

**Figure:**
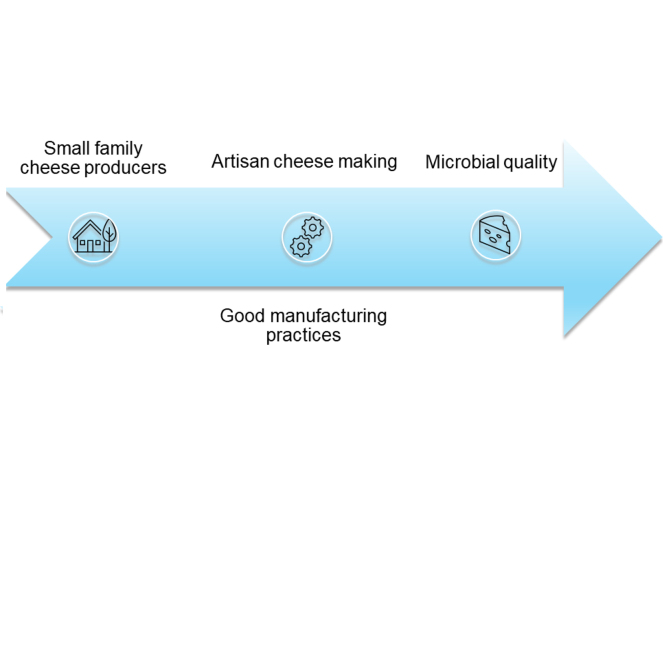
**Summary:** In this mini-review, we provide an overview of the microbial quality of artisan fresh raw milk cheese, including cheesemaking and hygienic practices of small family cheesemakers (SFC) in Mexico. Overall, SFC artisanal fresh cheeses had high microbial indicator populations and presence of foodborne pathogens such as *Salmonella*. However, Cocido cheese had low microbial populations and absence of both *Salmonella* and *Listeria* monocytogenes, despite being made with raw milk. Cheesemaking steps such as curd cooking may help to reduce the microbial load from raw milk, but may not be enough to ensure cheese safety. Limited or a lack of knowledge of hygienic practices is a contributing factor to microbial contamination of cheeses made by SFC. Training for SFC in good manufacturing practices has shown a positive effect on the microbial quality of raw milk and artisan cheese.

Dairy production is a significant economic activity in Mexico; more than 13 billion liters of milk and about 634 thousand metric tonnes of cheese were produced in 2023 with a combined market value greater than 194 billion Mex$ (∼10.4 billion US$; CANILEC, 2024; SIAP, 2025). An estimated 257,000 producers are dedicated to milk production in Mexico, from which 58% are small producers with fewer than 100 cows each (LICONSA, 2024). Among small dairy producers, also referred as production units, small family farms are an important production system in Mexico. Small family farms are generally characterized as family-owned farms with herds of up to 35 cows (Fadul-Pacheco et al., 2013), where labor is performed by family members (Hernandez-Velasquez et al., 2022), and milk production is either sold to nearby small cheese factories or processed at their farm to produce dairy products such as cheese (Cesín-Vargas et al., 2007). However, small family dairy farms owning smaller herds (e.g., 6 heads or fewer) are most likely to process the milk into dairy products, particularly cheese (Cesín-Vargas et al., 2007), which is more profitable than selling lower volumes of milk (Zepeda-Cancino and Vázquez-García, 2023). Additionally, the percentage of family farmers that produce cheese (i.e., cheesemakers) in certain rural regions in Mexico has been reported to be between 18% and 36% (Cesín-Vargas et al., 2007; Zepeda-Cancino and Vázquez-García, 2023).

Cheese consumers in Mexico have appreciation for artisan-made cheeses, as they are perceived as authentic, particularly for 2 main reasons: their sensory characteristics (compared with large-scale manufacturer-made cheeses) and their association with rurality (artisan-made in rural areas; Fernández-Sánchez et al., 2024). Artisanal cheeses made by small family cheesemakers (**SFC**) have a wide presence across the country; farmers may sell them home to home, at nearby towns' convenience stores, at public or street markets, or even from their own homes (Cesín-Vargas et al., 2007; Zepeda-Cancino and Vázquez-García, 2023). However, raw milk is typically used for artisanal cheesemaking, resulting in cheeses with a short shelf life of up to 1 wk (Cesín-Vargas et al., 2007) and bringing public health concerns due to the risk of foodborne pathogens.

In 2024, Mexico's National System of Epidemiology Surveillance (SINAVE) reported more than 4.3 million cases of foodborne illnesses (categorized as bacterial food poisoning and infectious intestinal diseases; SINAVE, 2024), but no official data regarding implicated food consumed in foodborne illness cases is available. Thus, the association of cheese consumption in Mexico, particularly artisanal cheeses, to foodborne pathogens is unknown. Although artisanal cheeses in Mexico, especially those made by SFC, are locally marketed in the country, artisanal Mexican cheeses may be introduced in other countries. For example, travelers may bring milk and dairy products from Mexico into the United States (USDA APHIS, 2024). *Salmonella* and *Listeria monocytogenes* have been isolated from cheeses, homemade or acquired from farms, carried by people crossing the US land border from Mexico (Kinde et al., 2007). Rodríguez-Lázaro et al. (2017) reported that one dairy product illegally entering the EU on a flight from Mexico tested positive for *Staphylococcus aureus*. The aforementioned studies highlight that artisanal cheeses in Mexico, when contaminated with foodborne pathogens, may affect public health in Mexico and other countries.

The presence of foodborne pathogens has been reported in different types of cheeses sold at street or public markets, common places where SFC sell the cheeses they manufacture. Camarillo et al. (2018) reported the presence of aflatoxin M1, ranging from 0.02 to 22.8 µg/kg, in Oaxaca cheese (also known as Quesillo) sold in small grocery stores in the state of Veracruz. Pathogens such as methicillin-resistant *S. aureus* (**MRSA**), enterotoxin A-producer *S. aureus* and *Bacillus cereus* have been found in Queso Fresco, Requeson, Cotija, Adobera, and Oaxaca cheeses (MRSA was not detected in Oaxaca cheese) that were marketed in street or public markets in the state of Guerrero (Adame-Gómez et al., 2018, 2020). Also, *Salmonella* was detected in Panela and Ranchero cheeses sampled from street markets in the state of Chihuahua, but no sampled cheeses were positive for *L. monocytogenes* (Chávez-Martínez et al., 2019).

Some consumers prefer to buy cheeses from street vendors or public markets due to the expectation that such artisanal cheeses were made by small farmers (Camarillo et al., 2018). However, artisanal raw milk cheeses made by small cheese factories (basic infrastructure facilities, family labor, no commercial brand, cheese production ∼20–100 kg/d) that are non-SFC can also be sold at street and public markets (Cesín-Vargas et al., 2007; Soria-Herrera et al., 2021). The commercialization of cheeses made by small factories in street and public markets may be higher due to their greater production compared with SFC cheeses. Therefore, not all cheeses sampled at street and public markets can be linked with certainty to SFC. Additionally, street or public markets in Mexico are generally installed on the street (open air), allowing free access to consumers, but most do not comply with health and safety regulations such as refrigeration (Chávez-Martínez et al., 2019). This may contribute to microbial contamination of marketed cheeses that may have had low microbial counts when initially manufactured.

A limited number of studies have investigated the microbial quality of artisanal cheeses made by SFC. The presence and population of yeast and molds, coliforms, *Escherichia coli*, *S. aureus*, *Salmonella*, and *L. monocytogenes* detected in cheeses sampled at small family farms located in the states of Sonora, San Luis Potosi, Zacatecas, and Baja California are shown in Table 1. Queso Fresco made by 18 SFC in Sonora state had the highest count range of indicator microorganisms and presence of *Salmonella* in 78% of the samples, whereas lower counts of indicator microorganisms and absence of *Salmonella* and *L. monocytogenes* were reported in Cocido cheese samples made from 6 SFC in Sonora state (Table 1). Queso Fresco and Cocido cheeses in those studies were reported to be made with raw milk (Cuevas-González et al., 2017; Méndez-Romero et al., 2021). However, differences in cheesemaking steps may have affected the microbial populations of the finished cheeses: Queso Fresco curd is generally uncooked (Ibarra-Sánchez et al., 2017), but cheesemaking of Cocido cheese requires a cooking step of the curd (50–60°C for 20 min), which significantly reduced the microbial load in the raw milk used (Cuevas-González et al., 2017). Rodríguez-Gallegos et al. (2022) reported that some SFC in the states of San Luis Potosi and Zacatecas “pasteurize” their milk (boiling the milk, then letting it cool down) for cheesemaking (Table 1). However, only 3 “pasteurized” goat milk cheeses and 3 raw cow milk cheeses showed low populations of total coliforms and *S. aureus*, but *Salmonella* was present in all cheeses sampled (raw and boiled milk cheeses). *Listeria monocytogenes* was not detected in samples tested in the studies displayed in Table 1. It is important to note that fresh cheeses made with either raw or pasteurized milk have been highly linked to *L. monocytogenes* outbreaks in the United States (Ibarra-Sánchez et al., 2017). Therefore, the potential risk of *L. monocytogenes* infection due to consumption of raw milk cheeses made by small family farmers exists.

**Table 1:** Microbial quality of artisanal fresh cheeses by small family cheesemaker farms in Mexico^1^

Cheese	State (production units)	Yeast/molds (log_10_ cfu/g)	Total coliforms (log_10_ cfu/g)	*Escherichia coli* (log_10_ MPN/g)	*Staphylococcus aureus* (log_10_ cfu/g)	*Salmonella* (positive samples/total samples)	*Listeria monocytogenes* (positive samples/total samples)	Reference
Queso Fresco	Sonora^2^ (18)	4.2–8.0	6.5–10.04 (log_10_ MPN/g)	6.38–10.04	3.66–6.14	14/18	0/18	Méndez-Romero et al., 2021
	San Luis Potosí^3^ (6)	NT	4.5–9.38	NT	2.36–6.92	18/18	NT	Rodríguez-Gallegos et al., 2022
	Zacatecas^2^ (6)	NT	2.87–10.41	NT	2.5–10.8	18/18	NT	Rodríguez-Gallegos et al., 2022
	San Luis Potosí^4^, ^5^ (3)	NT	<1–10.11	NT	<1–5.77	9/9	NT	Rodríguez-Gallegos et al., 2022
Cocido cheese	Sonora^2^ (6)	<1–2.6	1.6–3.0 (log_10_ MPN/g)	0.7–1.9	2.3–4.7	0/12	0/12	Cuevas-González et al., 2017
Ojos Negros pressed cheese	Baja California^2^ (22)	2.70–4.57	5.20 ± 0.18	NT	NT	0/22	0/22	Silva-Paz et al., 2020

1 MPN = most probable number; NT = not tested.

2 Raw milk used for cheesemaking by 100% production units.

3 Raw milk used for cheesemaking by 50% production units.

4 Raw milk used for cheesemaking by 66% production units.

5 Goat milk cheese.

In Mexico, the official standard NOM-243-SSA1-2010 (Secretaria de Salud, Estados Unidos Mexicanos, 2010) sets the permissible microbial limits for milk and dairy products such as fresh cheeses. However, NOM-243-SSA1-2010 establishes that milk must be pasteurized; therefore artisanal raw milk cheeses, particularly those made by SFC, are not included in NOM-243-SSA1-2010. Some microbial limits for fresh cheeses established in NOM-243-SSA1-2010 include yeast and molds (2.7 log_10_ cfu/g), *E. coli* (2 log_10_ cfu/g), *S. aureus* (3 log_10_ cfu/g), *Salmonella*, and *L. monocytogenes* (both absent/25 g). The studies listed in Table 1 indicate that few of the sampled cheeses made by SFC, particularly Cocido cheese, had acceptable microbial quality according to the NOM-243-SSA1-2010 microbial limits. The aforementioned studies (Table 1) suggest that not all type of cheeses made by SFC in Mexico have the same microbial risk, even though raw milk is used or, at best, heat treated (boiled) but not properly pasteurized. More studies conducted directly at small family dairy farms are needed to determine whether other raw milk artisanal cheeses have consistently acceptable microbial quality.

The cheesemaking experience of SFC in certain rural areas in Mexico has been reported to be between 10 and 40 years (Cesín-Vargas et al., 2007; Cervantes-Escoto et al., 2021; Hernandez-Velasquez et al., 2022). However, cheesemaking knowledge of SFC is mainly transmitted as empirical knowledge from one generation to another (Cervantes-Escoto et al., 2021). Therefore, understanding of good manufacturing practices (**GMP**) and the safety risks associated with the use of raw milk for cheesemaking may be limited. The cheesemaking processes followed by SFC, which differ between different types of cheeses, are critical to identify manufacturing steps that may increase risk of spoilage and pathogen contamination in the finished cheeses.

Cheesemaking steps that may influence the microbial quality of Cocido cheese include the use of commercial rennet, acidification of milk with fermented whey from a previous batch (a few may opt to add citric acid instead), and cooking the curd (50–60°C for 20 min; Cuevas-González et al., 2017). Rodríguez-Gallegos et al. (2022) reported that only 4 out of 15 SFC heat treat (boil) raw milk before cheesemaking of Queso Fresco. The same proportion of farmers (4/15) prefer to use commercial rennet, whereas the rest, including 3 farmers that heat treat raw milk, coagulate milk by adding natural rennet (1 farmer let natural rennet ferment before its use). In the case of Criollo cheese in cocoyam leaf, an artisanal cheese made by SFC in the state of Hidalgo, steps such as adding natural rennet to coagulate milk, shaping salted curd in wooden molds, and wrapping the final cheese in a cocoyam leaf (Cervantes-Escoto et al., 2021) may affect the microbial contamination of the cheese. However, the microbial quality of Criollo cheese in cocoyam leaf has not been studied. Particularly, the use of natural rennet for cheesemaking may contribute to microbial contamination of cheeses made by SFC. In Mexico, natural rennet is typically prepared by SFC using traditional methods. For example, calf stomach is first dried, followed by fermentation in milk whey at room temperature for several days. However, presence of coliforms, *E. coli*, *S. aureus*, and *Salmonella* has been reported in natural rennet prepared as described (Rendón-Huerta et al., 2023), suggesting microbial contamination risk of cheese made with natural rennet as coagulant.

Good manufacturing practices are a set of procedures and practices required to comply with GMP regulations, including facilities' construction, equipment and utensils, production and processing controls, and sanitary operations to produce safe food (FDA, 2024). Few studies have documented GMP during cheesemaking conducted by SFC in Mexico, including milking practices. It is estimated that less than 30% of small farms in Mexico have portable milking machines, with lack of economic resources and government financial aid limiting the incorporation of improved technology in traditional cheesemaking processes (Salinas-Martínez et a., 2020). Also, SFC have limited access to secondary preventative medicine programs for their herds, being unable to promptly detect and treat subclinical mastitis cases (Silva-Paz et al., 2020). Pathogens isolated from milk from clinical and subclinical cases of bovine mastitis include *Streptococcus dysgalactiae*, *Streptococcus uberis*, *S. aureus*, *E. coli*, and *Klebsiella pneumoniae* (Zheng et al., 2022; Sweeney et al., 2024). Recently, Boljkovac Begić et al. (2025) reported the presence of *L. monocytogenes* in raw milk semi-hard cheeses in Croatia, and the pathogen was traced back to the milk of a cow with chronic subclinical mastitis caused by *L. monocytogenes*. This suggests that clinical and subclinical mastitis not only compromises the health of the herd but also represents a risk for SFC families and the safety of the cheeses produced.

Regarding cheesemaking manufacturing and hygienic practices by SFC in Mexico, Rodríguez-Gallegos et al. (2022) reported that 2 SFC farms in the state of San Luis Potosi and 1 SFC farm in the state of Zacatecas had facilities dedicated exclusively to cheesemaking, and hygienic measures such as wearing hair caps, face masks, and boots, in addition to cleaning floors, equipment, and utensils used were observed. Utensils such as plastic or aluminum buckets, cloths (for milk filtering), and wooden or plastic molds (Cervantes-Escoto et al., 2021) are potential sources of microbial contamination if they are not properly cleaned and sanitized. Inadequate raw milk storage before cheesemaking can promote the growth of spoilage and pathogenic bacteria. Silva-Paz et al. (2020) observed that raw milk used for cheesemaking of Ojos Negros pressed cheese (Baja California state) was stored at room temperature (approximately 20°C). Additionally, direct manipulation of the curd with bare hands (e.g., salting, molding, and unmolding; Rangel-Ortega et al., 2024) could contribute to microbial contamination of the final cheese. Nonhygienic practices compromise the sanitary manufacture of artisanal cheeses, as food contact surfaces are potential sources of microbial contamination. For example, in small cheese processing facilities, *S. aureus* has been recovered from tables, knives, workers' hands, and hand washing basins (Castañeda-Ruelas et al., 2017; Ebied et al., 2022), and coliforms have also been detected on workers' hands and hand washing basins (Ebied et al., 2022).

Some SFC may attempt to incorporate a pasteurization step in their cheesemaking process, but lack of knowledge and proper training may result in improper pasteurization. Rodríguez-Gallegos et al. (2022) documented that 4 SFC in the state of San Luis Potosi “pasteurized” their raw milk (boiling the raw milk). However, such heat treatment did not have a significant improvement on the microbial quality of Queso Fresco produced compared with farmers who did not heat treat raw milk (Table 1), suggesting microbial contamination after heat treatment. Although milk pasteurization is effective to reduce the microbial load of raw milk and to kill pathogenic bacteria to ensure cheese microbial safety, SFC are less likely to consider pasteurizing their milk, not only due to changes in cheese texture and flavor, but also due to the additional cost (Cervantes-Escoto et al., 2021).

In Mexico, GMP regulations applicable to cheeses can be found in official standards NOM-251-SSA1-2009 (minimal requirements of good hygienic practices for food processing to reduce microbial contamination; Secretaria de Salud, Estados Unidos Mexicanos, 2009) and NOM-243-SSA1-2010 (sanitary requirements for milk and dairy products; Secretaria de Salud, Estados Unidos Mexicanos, 2010). Particularly, NOM-251-SSA1-2009 requires GMP training to food industry workers at least once a year, but rural food producers, such as SFC, are not included in the official standard. Currently, no official standard in Mexico establishes sanitary requirements for rural food producers. To aid rural farmers to improve their knowledge, practices, and productivity, Mexico's National Institute for Rural Sector Capacities Development (INCA RURAL) has been offering online training courses for small family dairy producers in Mexico, such as hygienic feeding and handling of dairy cattle (INCA RURAL, 2022). It is important to consider that only 4% to 16% of rural households in Mexico have internet access (García-Mora and Mora-Rivera, 2023); therefore such online training courses are expected to have limited reach.

Acquisition of GMP knowledge through training programs could help SFC to improve the microbial quality of the cheeses made. Flores-Herrera et al. (2014) implemented a good hygienic practices training program, emphasizing the importance of milk and water microbial quality and hygiene in the entire milking process, in 20 dairy farms that manufacture Cotija cheese in Michoacan state. At least 1 log_10_ cfu/mL reduction of both mesophilic aerobic bacteria and total coliforms in raw milk after training was observed. *Salmonella*, *E. coli*, and *S. aureus* were initially all detected in at least 90% of raw milk samples, but after training, less than 10% of raw milk samples were positive for these pathogens. However, that study was limited to training on good milking hygienic practices, and microbiological testing was performed only with raw milk. Thus, it is unclear the impact on the microbial quality on the final product (in this case, Cotija cheese). Further, it is unknown whether additional training on GMP in cheesemaking may be needed. Other studies have reported the influence of GMP training on the microbial quality of artisanal cheeses made in Latin America. Ramón et al. (2018) trained 26 aboriginal families in Argentina who produce goat milk cheese in GMP (e.g., GMP at milking and dairy processing, handlers' hygienic practices, importance of zoonoses and foodborne diseases). Before GMP training, some nonhygienic practices were observed: poor hygienic hand milking, no pasteurization equipment, dirty floor in production areas, wearing neither apron nor cap, and workers' habits not meeting hygienic standards. After training, producers improved their hygienic habits and practices: utensils sanitation, washing animals' udders, wearing apron and cap, milk pasteurization, milk temperature monitoring, and overall cheesemaking process standardization. Additionally, improvement of the microbial quality of the goat cheese, particularly total coliforms, was reported. The population of total coliforms in cheese before training was ∼7 log_10_ cfu/g, but after training, total coliform counts were ≤3.7 log_10_ cfu/g. These studies illustrate the positive effects of GMP trainings to SFC on the microbial quality of milk and artisanal cheese. However, this practice is still poorly implemented for SFC in Mexico, considering that GMP training programs may need to be adapted to SFC needs (e.g., lack of economic investment or inability to add pasteurization costs).

This mini-review describes the microbial quality of selected SFC artisanal cheeses, as well as cheese manufacturing process and GMP that affect microbial contamination of SFC artisanal fresh cheeses in Mexico. Most SFC artisanal cheeses had poor microbial quality, including high microbial indicator populations and presence of foodborne pathogens. However, Cocido cheese had acceptable microbial quality based on Mexican regulations, despite being made with raw milk. Although in general SFC do not pasteurize the milk used for cheesemaking, artisanal cheesemaking steps such as curd cooking could reduce the microbial load from raw milk, but it may not be enough to ensure the safety of raw milk cheese. Limited GMP knowledge is an important barrier to the safe production of cheese by SFC. Training courses on GMP, in addition to those offered by the Mexican government, are needed to increase the reach to more SFC residing in rural communities. Overall, further studies on microbial quality and manufacturing practices of SFC artisanal cheeses are needed to better understand microbial contamination risks during production, to ultimately develop strategies to effectively aid SFC in Mexico to improve artisanal cheese safety.
